# *Rice yellow mottle virus* is a suitable amplicon vector for an efficient production of an anti-leishmianiasis vaccine in *Nicotiana benthamiana* leaves

**DOI:** 10.1186/s12896-024-00851-8

**Published:** 2024-04-24

**Authors:** PKA Bamogo, F Tiendrébéogo, C Brugidou, D Sérémé, FW Djigma, J Simporé, S Lacombe

**Affiliations:** 1 Institut de L’Environnement et de Recherches Agricoles (INERA), LMI Patho-Bios Laboratoire de Virologie et de Biotechnologies Végétales, Ouagadougou, Burkina Faso; 2 Université Joseph Ki-Zerbo, Laboratoire de biologie moléculaire et de Génétique (LABIOGENE), Ecole Doctorale Sciences et Technologie, Centre de recherche biomoléculaire Piétro Annigoni (CERBA), Ouagadougou, Burkina Faso; 3 PHIM Plant Health Institute Montpellier, Univ Montpellier, IRD, CIRAD, INRAE, Institut Agro, Montpellier, France

**Keywords:** PSA, RYMV, Plant-based viral vector, Leishmaniosis

## Abstract

**Background:**

Since the 2000’s, plants have been used as bioreactors for the transient production of molecules of interest such as vaccines. To improve protein yield, “amplicon” vectors based on plant viruses are used. These viral constructs, engineered to carry the gene of interest replicate strongly once introduced into the plant cell, allowing significant accumulation of the protein. Here, we evaluated the suitability of the monocot-infecting RNA virus *Rice yellow mottle virus* (RYMV) as an amplicon vector. The promastigote surface antigen (PSA) of the protozoan *Leishmania* was considered as a protein of interest due to its vaccine properties against canine leishmaniasis.

**Results:**

Since P1 (ORF1) and CP (ORF3) proteins are not strictly necessary for viral replication, ORF1 was deleted and the PSA gene was substituted to ORF3 in the RYMV-based vector. We evaluated its expression in the best described plant bioreactor system, *Nicotiana benthamiana* which, unlike rice, allows transient transformation by *Agrobacterium*. Despite not being its natural host, we demonstrated a low level of RYMV-based vector replication in *N. benthamiana* leaves. Under optimized ratio, we showed that the P19 silencing suppressor in combination with the missing viral CP ORF significantly enhanced RYMV amplicon replication in *N. benthamiana*. Under these optimized CP/P19 conditions, we showed that the RYMV amplicon replicated autonomously in the infiltrated *N. benthamiana* cells, but was unable to move out of the infiltrated zones. Finally, we showed that when the RYMV amplicon was expressed under the optimized conditions we set up, it allowed enhanced PSA protein accumulation in *N. benthamiana* compared to the PSA coding sequence driven by the 35S promoter without amplicon background.

**Conclusion:**

This work demonstrates that a non-dicot-infecting virus can be used as an amplicon vector for the efficient production of proteins of interest such as PSA in *N. benthamiana* leaves.

**Supplementary Information:**

The online version contains supplementary material available at 10.1186/s12896-024-00851-8.

## Background

The use of plants as bioreactors for the production of proteins of interest such as therapeutic or industrial proteins has boomed in recent decades. An increasing variety of complex and valuable molecules of interest are now produced in plant bioreactors [1, 2]. Compared to traditional biological “factories”, which are mainly mammalian and microbial, plant bioreactors cannot be infected by mammalian pathogens. In addition, these alternative systems are cost-effective and easy to scale up. *Agrobacterium*-mediated transient transformation, called agroinfiltration, can be used to transiently produce recombinant proteins in intact leaves within a few days [3]. *Nicotiana* plant species such as *N. tabaccum* and *N. benthamiana* are preferred due to their high biomass, rapid growth rate and their ability to be stably and transiently transformed by *Agrobacterium* [2, 4, 5]. One of the major limitations in these plant based transient gene expression systems is the relatively limited product yield. Indeed, setting up production systems that lead to expression levels acceptable for economic production (> 50 mg/kg for antibodies) is not a trivial process. One of the explanations is the post-transcriptional RNA silencing that the plant cells establish in response to the introduction of foreign nucleic acids [6]. Co-expression of viral proteins that suppress RNA silencing activity overcomes this limitation and allows for increased levels of transient expression [7, 8]. Another effective advance in yield enhancement is also based on viruses. It involves the development of plant viral vectors that express proteins of interest in plants: amplicon technology. Once introduced into a host plant cell by agroinfiltration, a viral vector engineered to contain a gene of interest replicates and the corresponding protein can be rapidly produced in significant quantities [9, 10]. This method has been shown to work with many proteins. Viral vectors used so far were mostly derived from *Solanaceae* infecting viruses such as *Tobacco mosaic virus* (TMV) and *Potato virus X* (PVX) combined with host plants such as *N. tabaccum* and *N. benthamiana* [11–13]. The first generation of viral vectors was based on the addition of the gene of interest to the complete viral genome. However, a negative correlation between insert size and virus stability was observed, which could be due to the rapid loss of the transgene during viral replication [14, 15]. A more efficient generation of viral vectors has been based on the deconstruction of viral genome where only elements required for replication are retained. This allows the insertion of large genes of interest while maintaining viral genome size and stability [15, 16].

*Rice yellow mottle virus* is a member of the genus *Sobemovirus*. Its genome is a single-stranded positive-sense RNA molecule of approximately 4.5 kb. Its 5’ end has a covalently attached viral protein (VPg) and its 3’ end is not polyadenylated [17, 18]. The genome contains five protein-coding Open Reading Frames (ORFs). ORF1, x, 2a and 2b are translated from the genomic RNA while ORF3 is translated from the sub-genomic RNA [19]. Except for the recently identified ORFx [19], the functions of all other ORFs are well characterized. ORF1 encodes the P1 protein which has been described as a suppressor of RNA silencing [20–22]. P1 is also involved in viral movement [23]. ORF2a and 2b encode polyproteins that are cleaved to produce VPg, a serine protease, a helicase, and an RNA-dependent RNA polymerase (RdRp) [17, 18]. ORF3 encodes the Coat Protein (CP) [17]. Functional studies have shown that both P1 and CP are dispensable for virus replication in the rice protoplast [23, 24]. Using the RYMV infectious cDNA clone developed by [24], non-requirement of P1 for viral replication in rice plants was confirmed [25]. The natural hosts of RYMV are restricted to several species of the genus *Oryza* [26]. It is mechanically transmissible to rice, but also to several graminaceous species [27]. The RYMV dilution endpoint which is quite low (10^-11^), reveals significant virus titers in infected samples, suggesting strong viral stability and a high replication rate [26]. Using the RYMV infectious cDNA clone, viral replication was detected not only in rice but also in wheat, oat, and barley monocots and in *Arabidopsis thaliana* and *N. benthamiana* dicots [25]. Based on its relatively simple and well characterized genome, the unnecessary P1 and CP for viral replication, its high replication rate and its ability to replicate in *N. benthamiana*, RYMV represents a candidate of choice for the development of a new amplicon tool for the production of proteins of interest in *N. benthamiana*.

Here, we addressed this issue by developing an RYMV amplicon vector in which the P1 and CP genomic sequences not required for virus replication were removed. We focused on the Promastigote Surface Antigen (PSA) protein as the protein of interest to be produced. Indeed, this protein derived from the parasitic protozoan *Leishmania,* has been characterized as having efficient vaccine properties against canine leishmaniasis [28, 29]. Futhermore, we have previously shown that an active PSA protein can be transiently accumulated in *N. benthamiana* leaves after agroinfiltration of a PSA coding sequence driven by the constitutive 35S promoter. However, despite optimized RNA silencing suppression and subcellular localization, PSA accumulation reached only 0.4 mg/kg of fresh weight which is insufficient for economic production [7]. Here, we replaced the CP subgenomic coding sequence with the PSA sequence in the RYMV amplicon vector. A series of experiments were performed to optimize replication of the RYMV amplicon vector in *N. benthamiana* leaves, allowing for improved accumulation of the protein of interest. This work demonstrates that the RYMV amplicon tool and procedure developed here are effective for transient accumulation of proteins of interest in *N. benthamiana* in a rapid, efficient, and cost-effective manner.

## Results

### Design of RYMV amplicon vector

**Fig. 1:**
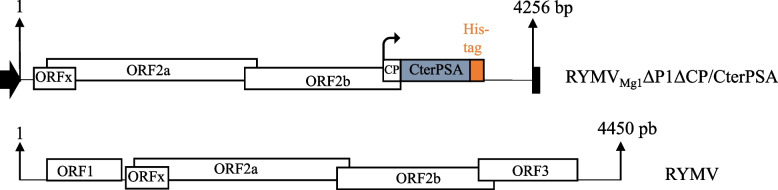
Schematic representation of the RYMV_Mg1_ΔP1ΔCP/CterPSA construct based on the RYMV genome. For both theRYMV_Mg1_ΔCPΔP1/CterPSA construct and the RYMV reference, ORFs are represented by white boxes. The sizes of both sequences are given. For the RYMV_Mg1_ΔCPΔP1/CterPSA construct, the insert encoding for the C-terminal portion of *Leishmania* PSA (CterPSA) is indicated by the blue box. A His tag, represented by the orange box, is fused to its C-terminal portion. Black arrow and black box represent the CaMV duplicated 35S promoter and CaMV polyA signal sequence / terminator, respectively

The construction of amplicon tools must minimize the modification of genome structure in order to avoid the expulsion of sequences encoding the protein of interest during viral replication. As a result, sequences of interest have to be inserted instead of viral sequences that are not necessary for viral replication. Previous works have shown that both P1 protein and CP, encoded by ORF1 and ORF3 respectively, are dispensable for virus replication in rice protoplasts and plants [23–25]. Both P1 and CP sequences were removed and the sequence of interest was inserted into the highly replicative subgenomic portion of the RYMV genome in place of CP ORF (Fig. 1). The 5’ end of CP ORF overlaps with ORF2b. In order to maintain the integrity of ORF2b coding sequence, CP ORF replacement has to be partial maintaining the ORF2b / CP ORF overlapping sequence. Consequently, a CP / protein of interest fusion is expected to be produced. The P1 protein has previously been described as a suppressor of the RNA silencing defense mechanism [20, 21]. It has been shown that the efficiency of RNA silencing suppression varies among RYMV isolates from weak to strong suppressors, such as P1 from the Madagascar isolate (Mg1) and P1 from the Tanzania isolate (Tz3), respectively [20, 21]. Therefore, we chose to consider the Mg1 genetic background for the amplicon tool developed here, as only a weak RNA silencing suppression activity would be missing (Fig. 1). The protein of interest was the PSA protein from the parasitic protozoan *Leishmania* which has anti-leishmaniasis vaccine properties. We previously succeeded in producing this protein in *N. benthamiana* using a 35S expression vector (35S::PSA) but failed to achieve an acceptable production yield [7]. Here, we considered only the carboxy-terminal part of PSA (CterPSA), as it induced the higher level of protection in tested dogs [30], and produced the resulting amplicon vector RYMV_Mg1_ΔP1ΔCP/CterPSA (Fig. 1).

### RYMV-based amplicon RNA accumulated efficiently in Nicotiana benthamiana with CP trans complementation

**Fig. 2:**
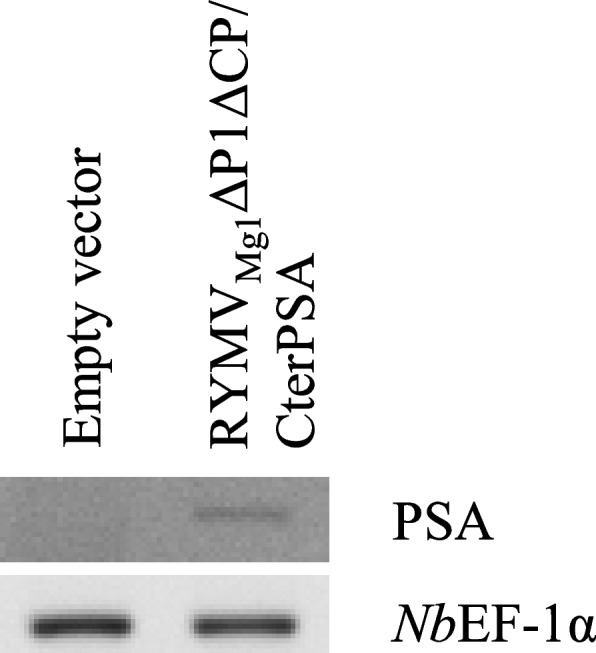
RYMV_Mg1_ΔP1ΔCP/CterPSA RNA accumulation in *Nicotiana benthamiana* leaves. Semi-quantitative RT-PCR was performed on RNA extracted from *N. benthamiana* leaves infiltrated with either empty vector or RYMV_Mg1_ΔP1ΔCP/CterPSA construct together with the cocktail of RNA silencing suppressors. Reverse transcriptions were performed using a mixture of oligodT and CterPSA specific primers. PCR amplification was performed using CterPSA-specific primers. *Nb*EF1-α expression was used as internal control. Three independent experiments were performed. The results presented here are representative of the three experiments

Under natural conditions, RYMV hosts are restricted to grass species such as rice. We first investigated whether RYMV_Mg1_ΔP1ΔCP/CterPSA RNA accumulates in *N. benthamiana* leaves. We used an optimized RNA silencing suppression condition that has previously been shown to favor mRNA accumulation of the gene of interest in transient *N. benthamiana* assays [7]. We co-infiltrated *N. benthamiana* with a 1:1 ratio of the RYMV_Mg1_ΔP1ΔCP/CterPSA construct and the cocktail of RNA silencing suppressors described in [7]. The RNA silencing suppression cocktail consists of a 1:1:1 combination of three viral suppressors (P0 from *Beet western yellows virus*, P1 from RYMV, and P19 from *Cymbidium ring spot virus*) that act on different steps of the RNA silencing pathway. The empty vector was used as a negative control. Infiltrated leaves were harvested four days post infiltration (4 dpi) for total RNA extraction followed by semi-quantitative RT-PCR of the CterPSA sequence. As expected, no amplification was observed for the empty vector control samples. Low amplification was observed for the RYMV_Mg1_ΔP1ΔCP/CterPSA samples (Fig. 2). This indicated that the RYMV_Mg1_ΔP1ΔCP/CterPSA construct can be transcribed when expressed in *N. benthamiana* leaves but the corresponding RNA accumulated poorly even under optimized RNA silencing suppression conditions.

**Fig. 3:**
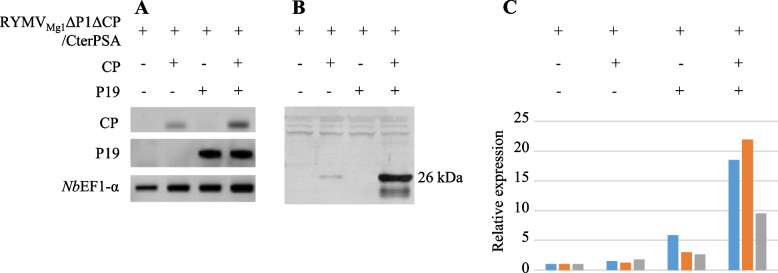
P19 RNA silencing suppressor and CP act synergistically to enhance RYMV_Mg1_ΔP1ΔCP/CterPSA RNA accumulation. *N. benthamiana* leaves were infiltrated with the RYMV_Mg1_ΔP1ΔCP/CterPSA construct alone (RYMV_Mg1_ΔP1ΔCP/CterPSA : empty vector 1:1 ratio) or with CP and P19 constructs either alone (RYMV_Mg1_ΔP1ΔCP/CterPSA:CP or P19:empty vector 1:0,5:0,5 ratio) or together (RYMV_Mg1_ΔP1ΔCP/CterPSA:CP:P19 1:0,5:0,5 ratio). Infiltrated leaves were harvested at 5 dpi. Three independent experiments were carried out. **A-** Semi-quantitative RT-PCR was performed after reverse transcription of RNA using with a mixture of oligodT and CterPSA specific primers. PCR amplifications was performed with CP and P19 specific primers. *Nb*EF1-α expression was used as an internal control. The results shown here are representative of the three experiments. **B-** Total soluble proteins were prepared from the same *N. benthamiana* samples. Equal amounts of proteins (10 µg) were separated by SDS-PAGE and analyzed by Western immunoblotting using anti-CP antibody. The size of the CP signals is indicated in kDa. The results shown are representative of the three experiments. **C-** RYMV_Mg1_ΔP1ΔCP/CterPSA RNA accumulation was evaluated by q-RTPCR for cDNA samples prepared in A. CterPSA-specific primers were used. Normalization was performed using GAPDH as a housekeeping internal control. Expressions were evaluated relative to the “RYMV_Mg1_ΔP1ΔCP/CterPSA alone” Condition. The three independent experiments are represented by different colors

P1 and CP have been described as dispensable for virus replication in rice protoplasts and plants. However, we cannot exclude that they may be required for efficient replication of the RYMV_Mg1_ΔP1ΔCP/CterPSA amplicon in *N. benthamiana* leaves. This does not appear to be the case for P1, as its presence in the RNA silencing suppressor cocktail used previously resulted in only weak accumulation of RYMV_Mg1_ΔP1ΔCP/CterPSA RNA (Fig. 2). We previously reported that replication of an RYMV infectious clone lacking the CP genomic sequence is optimized by the addition of an RYMV helper that complements the infectious clone with CP [31]. To test the effect of CP *trans*-complementation in N. *benthamiana*, a 35S-driven CP construct was co-infiltrated together with RYMV_Mg1_ΔP1ΔCP/CterPSA into *N. benthamiana* leaves at a ratio of RYMV_Mg1_ΔP1ΔCP/CterPSA:CP:empty vector of 1:0.5:0.5. CP RNA and protein accumulation were evaluated by semi-quantitative RT-PCR and Western blot, respectively (Fig. 3A, 3B). Signals were detected at both RNA and protein levels, but quite weak. Since CP is expressed as an exogenous gene, its expression should be affected by the RNA silencing defense pathway. To test this, RYMV_Mg1_ΔP1ΔCP/CterPSA and CP constructs were co-inoculated with P19 RNA silencing suppressor in a ratio of 1:0.5:0.5 (amplicon:CP:P19). Semi-quantitative RT-PCRs and Western blots show that the presence of P19 enhances both CP RNA and protein accumulation indicating that P19 protects CP from RNA silencing degradation (Fig. 3A, 3B). RYMV_Mg1_ΔP1ΔCP/CterPSA RNA accumulation was followed by q-RT-PCR for all tested conditions with or without CP and/or P19 (Fig. 3C). Enhanced amplicon RNA accumulation was only observed when both CP and P19 were present. This suggests that the enhanced CP accumulation due to P19 increases by *trans*-complementation, RYMV_Mg1_ΔP1ΔCP/CterPSA RNA accumulation in *N. benthamiana*.

### Optimization of RYMVMg1ΔP1ΔCP/CterPSA RNA accumulation in N. benthamiana

**Fig. 4:**
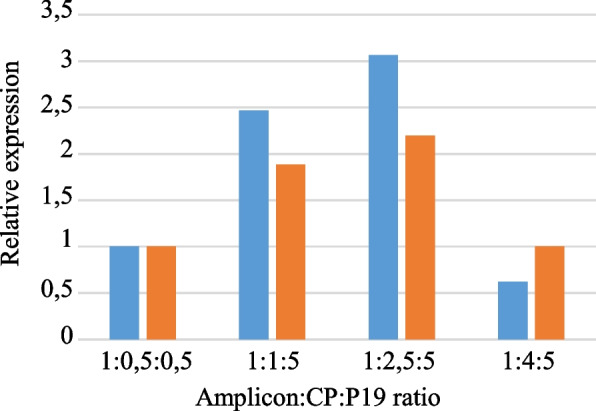
Optimal CP:P19 ratio for RYMV_Mg1_ΔP1ΔCP/CterPSA RNA accumulation. *N. benthamiana* leaves were infiltrated with the RYMV_Mg1_ΔP1ΔCP/CterPSA construct together with CP and P19 constructs. Four different amplicon:P19:CP ratios were considered. Infiltrated leaves were harvested at 5 dpi. Two independent experiments were performed. q-RT-PCR was performed after reverse transcription of RNA using a mixture of oligodT and CterPSA specific primers. PCR amplification was performed with CterPSA-specific primers. GAPDH was used as an internal housekeeping control. For each ratio, expressions were evaluated relative to the baseline ratio of 1:0,5:0,5. The two independent experiments are represented by different colors

To evaluate whether increasing the amount of CP and P19 could improve their positive effect on RYMV_Mg1_ΔP1ΔCP/CterPSA RNA accumulation, the initial 1:0.5:0.5 (amplicon:CP:P19) ratio was compared to three other ratios with increased amounts of CP and P19 constructs (Fig. 4). RYMV_Mg1_ΔP1ΔCP/CterPSA RNA accumulation was followed by q-RT-PCR. Results showed that increasing CP and P19 amount up to a ratio of 1:2.5:5 ratio (amplicon:CP:P19) improved RYMV_Mg1_ΔP1ΔCP/CterPSA RNA accumulation compared to the initial 1:0.5:0.5 condition. Beyond this threshold, a 1:4:5 ratio has a negative effect with a reduction in RYMV_Mg1_ΔP1ΔCP/CterPSA RNA accumulation compared to the initial 1:0.5:0.5 condition (Fig. 4). Consequently, we retained 1:2.5:5 (amplicon:CP:P19) ratio as the optimal one for amplicon RNA accumulation in *N. benthamiana* leaves.

**Fig. 5:**
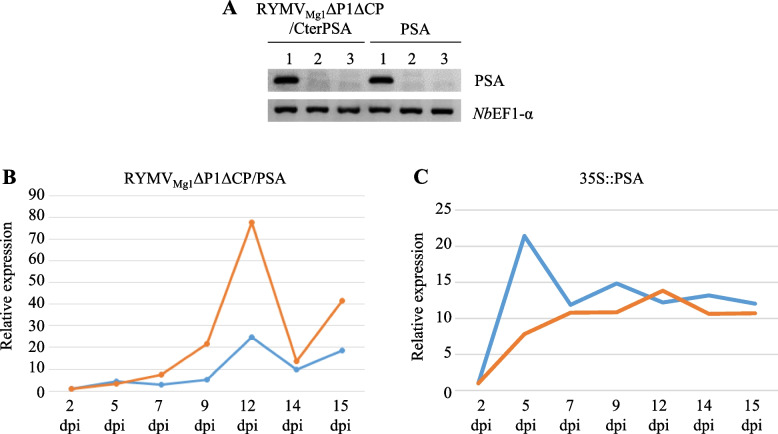
Spacio-temporal accumulation pattern of RYMV_Mg1_ΔP1ΔCP/CterPSA RNA. *N. benthamiana* leaves were infiltrated with RYMV_Mg1_ΔP1ΔCP/CterPSA construct together with CP and P19 constructs under the optimal 1:2,5:5 amplicon:CP:P19 ratio. 35S::PSA construct in combination with P19/P1/P0 RNA silencing suppressor cocktail was used as control. Two independent experiments were performed. **A-** At 5 dpi, RNAs were prepared from infiltrated area (1), adjacent non-infiltrated area of the same leaves (2) and systemic leaves (3). Semi-quantitative RT-PCR was performed after reverse transcription using a mixture of oligodT and CterPSA specific primers. PCR amplification was performed with CterPSA specific primers. *Nb*EF1-α expression was used as an internal control. For both RYMV_Mg1_ΔP1ΔCP/CterPSA RNA **B** 35S::PSA **C** constructs, RNA accumulation was followed from 2 to 15 days post-inoculation (dpi) in the infiltrated area. q-RT-PCR was performed after RNA reverse transcription using a mixture of oligodT and CterPSA specific primers. PCR amplification was performed with CterPSA specific primers. Normalizations were performed using GAPDH used as an internal housekeeping control. Expressions were evaluated relative to the 2 dpi time point. The two independent experiments are represented by different colors

Once present in host cells, viruses replicate and move from cell to cell and through phloem transport to invade systemic tissues. To determine the tissues with the best amplicon RNA accumulation, we evaluated the ability of the RYMV_Mg1_ΔP1ΔCP/CterPSA amplicon to move through *N. benthaniama* tissues under optimal conditions of 1:2.5:5 ratio (amplicon:CP:P19). Infiltrated (1), adjacent (2) and systemic sink (3) tissues were considered to evaluate amplicon RNA accumulation by semi-quantitative RT-PCR (Fig. 5A). For both 35S::PSA control and RYMV_Mg1_ΔP1ΔCP/CterPSA constructs, PSA RNA accumulation was detected only in infiltrated areas, indicating that the RYMV_Mg1_ΔP1ΔCP/CterPSA construct is unable to move from cell to cell and by phloem transport. We then followed PSA RNA accumulation over time by q-RT-PCR to determine the stages with the best RNA accumulation (Fig. 5B, 5C). Both RYMV_Mg1_ΔP1ΔCP/CterPSA repeats showed a weak accumulation until 9 dpi and then a strong increase until 12 dpi followed by a decrease (Fig. 5B). The 35S::PSA control construct behaved differently showing a strong induction of accumulation from 2 dpi to 5 – 7 dpi and then a stabilization (Fig. 5C). Based on these results, we retained infected areas at 12 dpi with a ratio of 1:2.5:5 ratio (amplicon:CP:P19) as conditions with optimal RYMV_Mg1_ΔP1ΔCP/CterPSA RNA accumulation.

### RYMVMg1ΔP1ΔCP/CterPSA replicated autonomously in N. benthamiana leaves

**Fig. 6:**
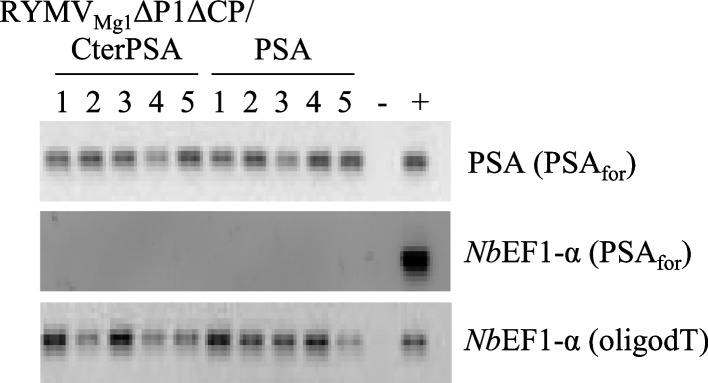
Accumulation of RYMV_Mg1_ΔP1ΔCP/CterPSA antisense RNA. *N. benthamiana* leaves were infiltrated with RYMV_Mg1_ΔP1ΔCP/CterPSA construct together with CP and P19 constructs under the optimal ratio of 1:5:2,5 amplicon:P19:CP. The35S::PSA construct in combination with the P19/P1/P0 RNA silencing suppressor cocktail was used as a control. Five individual plants, numbered from 1 to 5 were considered from both constructs. RNA was prepared from leaves infiltrated at 7 dpi. Reverse transcription was performed using either PSA primers hybridizing to the minus strand of the Cterm part of the PSA sequence (PSA_for_) or oligodT primers. The primer used for reverse transcription is indicated in parentheses. PCR amplification was performed using PSA-specific primers or *Nb*EF1-α primers. Negative (-) and positive (+) PCR controls were used

Once introduced into plant cells, the amplicon construct under the constitutive promoter is expected to first be transcribed as mRNA in the host cell nucleus to produce viral proteins required for viral replication, such as the viral RdRP. Then, autonomous viral replication can occur to amplify viral RNA through the synthesis of double-strand RNA mediated by viral RdRP. Therefore, to demonstrate that RYMV_Mg1_ΔP1ΔCP/CterPSA autonomous replication occurred, we searched for the accumulation of antisense RNA in *N. benthamiana* leaves infiltrated with RYMV_Mg1_ΔP1ΔCP/CterPSA construct under the previously determined optimal conditions. The 35S::PSA construct was used as a negative control. To detect antisense amplicon RNA, reverse transcriptions were performed with a PSA primer hybridizing to negative strand RNA (PSA_for_) followed by PCR amplification with PSA-specific primers (Fig. 6). PCR amplifications were detected for all RYMV_Mg1_ΔP1ΔCP/CterPSA samples. Surprisingly, amplifications were also detected in 35S::PSA negative control samples, suggesting possible DNA contamination. However, no amplifications occurred with *Nb*EF1-α primers on these antisense cDNA samples, whereas *Nb*EF1-α primers allowed amplifications on cDNA populations produced with oligodT primer (Fig. 6). These observations allow us to exclude a possible DNA contamination in antisense cDNA samples. Once introduced into *N. benthamiana* cells, both RYMV_Mg1_ΔP1ΔCP/CterPSA and 35S::PSA constructs are recognized as foreign nucleic acids and induce the RNA silencing defense mechanism. During this process, antisense RNA is produced from foreign RNA by an endogenous RdRP. Double-stranded RNA is then processed by Dicer enzyme to produce 21, 24 nt siRNA [6]. The RNA silencing suppressors used in our experiments (P19 alone or in combination of P1 and P0) act downstream of the double strand RNA step. This could explain the PSA PCR amplification in antisense cDNA samples for the 35S::PSA negative control. Therefore, in our transient expression system, the detection of antisense RNA is not an evidence of RYMV_Mg1_ΔP1ΔCP/CterPSA autonomous replication, since it can be produced by either viral or endogenous RpRPs.

**Fig. 7:**
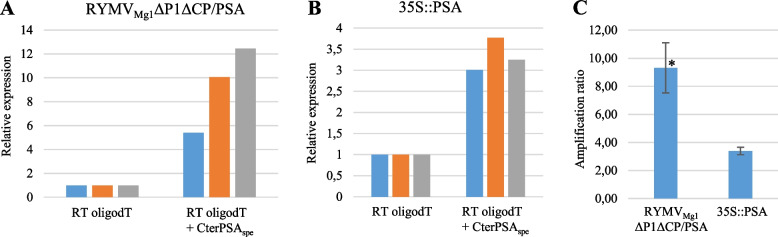
Comparison of total RNA accumulation with polyA mRNA accumulation. Total RNA previously prepared from *N. benthamiana* leaves infiltrated with either RYMV_Mg1_ΔP1ΔCP/CterPSA or 35S::PSA constructs was reverse transcribed using either oligodT or a mixture of oligodT and CterPSA specific primer (CterPSA_spe_). Three of the five individual plants were considerate. For both RYMV_Mg1_ΔP1ΔCP/CterPSA **A** and 35S:PSA **B** samples, q-RT-PCR was performed using CterPSA-specific primers. Normalizations were performed using GAPDH as an internal housekeeping control. For each construct, expressions were evaluated relative to the initial RT oligodT condition. The three independent plants are represented by different colors. **C-** The ratio of total RNA to mRNA accumulation was compared between RYMV_Mg1_ΔP1ΔCP/CterPSA and 35S::PSA samples. * Student t-test *p*=0,001

It has been shown that the genome of viruses such as RYMV, a member of the *Sobemovirus* family, is a single-stranded RNA with a non-polyadenylated (non-polyA) 3’ end [18]. Therefore, if the RYMV_Mg1_ΔP1ΔCP/CterPSA amplicon is autonomously active, a mixture of polyA and non-polyA RNA originating from host cell nuclear transcription driven by the constitutive promoter and from autonomous viral replication, respectively, should accumulate in infiltrated *N. benthamiana* cells. To assess whether non-polyA RNA accumulated, two cDNA populations were generated. The first produced by reverse transcriptions with the oligodT primer, represented the polyA mRNA population only. The second, generated by reverse transcriptions with a mixture of oligodT and CterPSA-specific primers (CterPSA_spe_), represented both polyA mRNA and non-polyA RNA populations, if present. q-RT-PCR on the CterPSA region was performed on these two cDNA populations for both RYMV_Mg1_ΔP1ΔCP/CterPSA and 35S::PSA samples previously obtained. Expressions for cDNA populations representing polyA plus non-polyA RNA were evaluated relative to expression for the cDNA population representing polyA mRNA only (Fig. 7A, 7B). For both RYMV_Mg1_ΔP1ΔCP/CterPSA and 35S::PSA samples, q-RT-PCR signals were stronger for polyA plus non-polyA cDNA populations compared to polyA ones. In the case of 35S::PSA samples, these increased q-RT-PCR signals could not be explained by an accumulation of non-polyA RNA, but rather by an improved reverse transcription efficiency with the CterPSA_spe_ primer compared to oligodT. In the case of RYMV_Mg1_ΔP1ΔCP/CterPSA samples, this increased amplification is significantly stronger than in the case of 35S::PSA samples (Fig. 7C). This suggests that it is not only due to the improved RT efficiency with the CterPSA_spe_ primer, but also to an accumulation of non-polyA RNA that characterizes an effective autonomous RYMV_Mg1_ΔP1ΔCP/CterPSA replication.

### RYMV_Mg1_ΔP1ΔCP/CterPSA allows enhanced CterPSA protein accumulation in infiltrated *N benthamiana* leaves

**Fig. 8:**
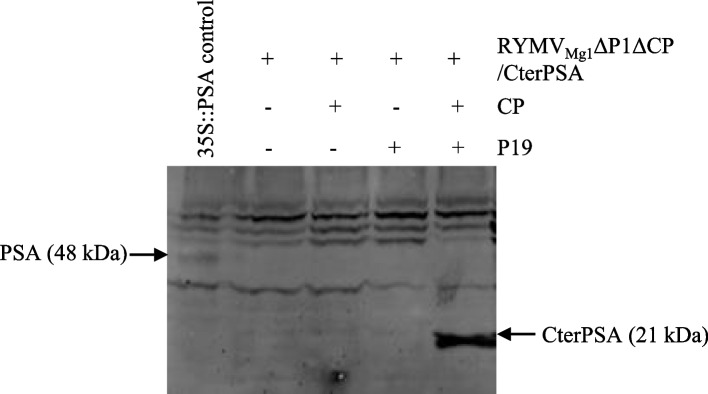
RYMV_Mg1_ΔP1ΔCP/CterPSA produces CterPSA protein under optimized conditions *N. benthamiana* leaves were infiltrated with RYMV_Mg1_ΔP1ΔCP/CterPSA construct alone (RYMV_Mg1_ΔP1ΔCP/CterPSA : empty vector 1:1 ratio) or with CP and P19 constructs either alone (RYMV_Mg1_ΔP1ΔCP/CterPSA:CP or P19:empty vector 1:0,5:0,5 ratio) or together (RYMV_Mg1_ΔP1ΔCP/CterPSA:CP:P19 1:2,5:5 ratio). Infiltrated areas were harvested at 12 dpi. The 35S::PSA construct in combination with the P19/P1/P0 RNA silencing suppressor cocktail was used as a control under the same conditions as described [7]. Total soluble proteins were prepared. Equal amounts of proteins (100 µg) were separated by SDS-PAGE and analyzed by immunoblotting using an anti-PSA antibody. Bands corresponding to PSA constructs and their corresponding sizes are indicated. Three independent experiments were performed. The results shown here are representative of the three experiments

To evaluate whether the RYMV_Mg1_ΔP1ΔCP/CterPSA construct is able to accumulate the expected protein of interest, CterPSA accumulation was evaluated by Western blot in *N. benthamiana* samples infiltrated with the RYMV_Mg1_ΔP1ΔCP/CterPSA construct. The amplicon construct was infiltrated on its own, with either CP or P19, and with both CP and P19 under previously established optimal conditions. The 35S::PSA construct was used as a control as described in [7] (Fig. 8). As expected, control samples accumulated 48 kDa PSA protein. For RYMV_Mg1_ΔP1ΔCP/CterPSA samples, CterPSA accumulation was only detected in samples expressing both CP and P19 at the expected 21 kDa size. This confirms at the protein level the requirement of P19-mediated CP *trans*-complementation for RYMV amplicon accumulation. Interestingly, the intensity of the Western signal was stronger for RYMV_Mg1_ΔP1ΔCP/CterPSA samples under optimal conditions compared to the 35S::PSA control sample (Fig. 8). Pixel signal quantification using ImageQuant TL software showed a sevenfold increase in intensity in the RYMV_Mg1_ΔP1ΔCP/CterPSA sample compared to the 35S::PSA control sample This confirms previous findings that RYMV_Mg1_ΔP1ΔCP/CterPSA is able to replicate autonomously once introduced into *N. benthamiana* cells under optimal conditions and that this active replication greatly enhances the accumulation of the protein of interest.

## Discussion

In the present work, we have shown that the RYMV genome can be used as an amplicon vector for the production of the protein of interest in *N. benthamiana* leaves under optimized conditions. Under natural conditions, RYMV infects only a few *Oryza* species. Using experimental mechanical transmission, it can infect several graminaceous species [26, 27]. Recently, [25] demonstrated that RYMV infectious clones also replicated in *N. benthamiana* and *A. thaliana* after biolistic delivery of RNA obtained by *in vitro* transcription. Here, we could not base the RYMV amplicon tool on infectious clones because *in vitro* transcription and biolistic delivery are not easily scalable, quite expensive, and therefore not compatible for our purpose. Using a 35S expression vector and *Agrobacterium tumefaciens* for delivery into *N. benthamiana* cells, we demonstrated that our RYMV amplicon tool is able to replicate autonomously. Our data, together with those of [25], suggest that even though *N. benthamiana* is not a RYMV host, its cells contain the machinery necessary for RYMV replication.

Using RYMV infectious clone mutants unable to express CP, [24] showed that CP is dispensable for virus replication in rice protoplasts. However, mutant replication was greatly reduced in rice plants compared to the wild-type infectious clone [24]. Our data confirm this point, as we showed only a weak replication of the amplicon construct lacking CP when expressed alone in *N. benthamiana* leaves. However, in our case we replaced the CP coding sequence with the CterPSA sequence, resulting in an altered genomic structure compared to the wild-type virus. Therefore, the stability of the amplicon could be affected, leading to a weak RNA accumulation. However, we showed that concomitant accumulation of CP at the protein level improved amplicon replication. This suggests that the amplicon-modified genomic structure does not affect RNA stability and that CP is required for efficient viral replication. This is supported by the fact that RYMV amplicon RNA is not detected outside the infiltrated zone because CP is only present in infiltrated cells. It could be interesting to evaluate whether CP-stable transgenic transformation of *N. benthamiana* could allow RYMV amplicon movement out of the infiltrated zone, as CP would constitutively accumulate in all tissues and consequently lead to improve accumulation of the protein of interest.

In the expression system developed here, we have shown that detection of antisense RNA is not an accurate way to demonstrate that the RYMV amplicon is replicating autonomously in *N. benthamiana* cells, since it can be produced by either viral or endogenous RdRP. In [25], the detection of antisense RNA allowed them to infer true viral replication. However, in their case, infectious clone constructs are introduced into *N. benthamiana* cells by biolistic RNA delivery, which allow integration of only a few RNA molecules bound to gold particles into the touched cells. This amount may not be sufficient to induce RNA silencing defense mechanism. Therefore, the detected antisense RNA could not be produced by the endogenous RdRP, but rather by the viral RdRP, in this case demonstrating autonomous viral replication.

*Cowpea mosaic virus* (CPMV) is widely used as a highly efficient amplicon system for the production of proteins of interest in *N. benthamiana* [32, 33]. Like RYMV, its natural host is not *N. benthamiana* but cowpea and it can infect other members of *Fabaceae* family [34]. One of the most important steps for the use of CPMV as an expression system was the creation of a 35S vector that could be inoculated in *N. benthamiana* by agroinfiltration [35]. Based on the work presented, RYMV represents a new non-*Solanaceae* efficient viral expression system for the production of proteins of interest in *N. benthamiana*.

## Conclusion

In this study, we reported successful development of a new RYMV-based viral expression vector using an optimized *N. benthamiana* transient expression system. We have shown that this RYMV-based vector, when complemented with a combination of P19 RNA silencing suppressor and RYMV CP, is highly suitable for the production of an anti-leishmaniasis vaccine candidate. This work will serve as a basis for further production of proteins of interest in *N. benthamiana*.

## Material and methods

### Plant expression vectors

Constructs for transient expression of empty vector, PSA, P19, P0 and P1 were as previously described in [7]. They correspond to pBIN61 binary vectors under the 35S promoter. CP coding sequence was prepared from cDNA population obtained from rice leaves infected by RYMV Mg1 isolate (provided by Agnès Pinel-Galzy) using RYMV specific primer (5’-CTCCCCCACCCATCCCGAGA-3’). PCR amplification was performed with CP Mg1 specific primers with *XbaI*/*HindIII* extension (in bold): CP_for_ 5’-GA**AAGCTT**ATGGCCAGGAAGGGCAAGAA-3’ and CP_rev_ 5’-GA**TCTAGA**TCACGTATTGAGTGTTGGAT-3’. PCR product was cloned into the 35S cassette of a pCambia1300 vector as described in [21]. RYMV_Mg1_ΔP1ΔCP/CterPSA was designed based on the RYMV sequence of Mg1 isolate (Genebank accession N° AJ608210). Sequences encoding P1 and CP proteins were removed. The sequence corresponding to the carboxy-terminal part of *Leishmaniana* Promastigote Surface Antigen protein (CterPSA) described in [30] was added instead of the CP sequence. The designed RYMV_Mg1_ΔP1ΔCP/CterPSA sequence flanked by the 35S promoter and terminator with *Kpn*I and *Pst*I, 5’ and 3’ ends, respectively, was synthesized *de novo* (Genecust, France). The corresponding sequence can be found in Additional file 1. RYMV_Mg1_ΔP1ΔCP/CterPSA sequence was then cloned into the pCambia1300 based expression vector at the *Kpn*I/*Pst*I sites as described in [21].

### *Agrobacterium tumefaciens*-mediated transient expression assay

The pBIN61 and pCambia1300 based constructs were introduced into either *A. tumefaciens* C58C1 or *A. tumefaciens* GV3101 strains, respectively. *N. benthamiana* plants were grown in a growth chamber at 12 h of light per day, 24 °C, and 60% relative humidity. Agroinfiltrations into 4-week-old *N. benthamiana* leaves were performed as described previously [36]. Briefly, recombinant *A. tumefaciens* strains were grown separately from precultures overnight at 28 °C and 200 rpm in an orbital shaker using LB medium containing rifampicin (100 μg/mL) and kanamycin (50 μg/mL). The cultures were pelleted by centrifugation at 4000 *g* for 10 min and the pellets were resuspended in 10 mM MgCl_2_ to a final OD600 of 0.5. Acetosyringone (4-hydroxy-3,5-dimethoxyacetophenone) was added to each suspension to a final concentration of 100 μM for virulence induction. Suspensions were incubated at room temperature for 4 h. Cultures were co-injected into *N. benthamiana* leaves at different ratios as indicated in Results section. For each experiment, at least two independent trials were performed to evaluate the reproducibility of the results.

### Semi-quantitative and quantitative RT-PCR

Total RNA was isolated from *N. benthamiana* leaves using TriReagent according to the supplier’s recommendations (Sigma-Aldrich). RNA samples were treated with RQ1 DNAse (Promega, Madison, Wisconsin, USA) and quantified using a Nanodrop spectrophotometer (Thermo Fisher Scientific, Waltham, Massachusetts, USA). cDNA preparations were made by reverse transcription using MMLV reverse transcriptase (Promega) with CterPSA specific primer (PSA_rev_: 5’-CAGGCACGTCCTCTCGTCCGTC-3’ ) alone or with oligodT primers as mentioned in the Results section. For antisense amplicon detection, reverse transcriptions were performed with a mixture of oligodT and PSA forward primers (PSA_for_: 5’-TGCTGGTGCGGACTGC-3’). Quantitative RT-PCR was performed using the Takyon Low Rox Sybr kit according to the manufacturer’s recommendations (Eurogentec, Liège, Wallonia, Belgium). PSA_for_ and PSA_rev_ primers were used for CterPSA PCR amplification. For semi-quantitative RT-PCR, the housekeeping gene encoding *Nb*EIF1-α (eukaryotic translation initiation factor 1-α from *N. benthamiana*) was used as an internal control with the primer pair F_*Nb*EF1-α_ (5’-TCACATCAACATTGTGGTCATTGGC-3’) and R_*Nb*EF1-α_ (5’-TTGATCTGGTCAAGAGCCTCAAG-3’). For q-RT-PCR, the *GAPDH* gene was used as normalization gene with the primer pair F_GAPDH_ (5-’GGTGTCAAGCAAGCCTCTCAC-3’) and R_GAPDH_ (5’-GATGCCAAGGGTGGAGTCAT-3’). The efficiency of the PCR for each technical replicate was validated using LinReg software [37]. The relative expression of the different genes targeted by qPCR was calculated according to the 2^-ΔΔCT^ method [38].

## Western blot

Transformed *N. benthamiana* leaves were ground with a mortar in liquid nitrogen for total soluble protein extraction. The equivalent of 1 mL of ground leaf powder was transferred to a 2 mL Eppendorf tube containing 300 µL of extraction buffer (20 mM Tris-HCl, 100 mM NaCl, 10 mM Na_2_EDTA 2H_2_O, 25 mM D-glucose, 0.1% Triton, 5 mM EGTA, 5% glycerol, 5 mM DTT). After centrifugation at 30 000 *g* for 15 min, the supernatants containing total soluble proteins were transferred to new microtubes. Protein concentration was determined using the Coomassie Plus Protein Assay (Thermo Scientific). Equal amounts of total soluble proteins (10 µg and 100 µg for CP and PSA detection, respectively) were separated on a 15% (w/v) SDS polyacrylamide gel. After migration, proteins were transferred to a Hybond-P membrane (RPN303F, GE Healthcare, Chicago, Illinois, USA.) using the Biorad Trans-Blot Turbo system. Membranes were incubated in TBS (20 mM Tris, 75 mM NaCl, and 2.5 mM MgCl_2_, pH 7.6) with 3% (w/v) milk powder for 1 h at room temperature to block non-specific binding. To detect RYMV CP, a rabbit polyclonal antibody (Sigma Aldrich, St. Louis, Missouri, USA.) was used at a dilution of 1:5000 (v/v) in TBS + 3% (w/v) milk powder. As described in [7], a rabbit polyclonal antibody raised against *Leishmania amazonensis* excreted/secreted antigen was used for PSA detection. This antibody was used at a dilution of 1:250 (v/v) in TBS + 3% milk powder (w/v). After washing with TBS, a peroxidase-linked secondary antibody anti-rabbit IgG (Immunopure Pierce Chemical, Dallas, TX, USA) was used at a dilution of 1:10000 in TBS + 3% milk powder (w/v) for 1 h at room temperature. After washing with TBS, detection was performed using the ECL Plus Western Blotting Detection System (GE Healthcare) with the ChemiDoc imaging system from Biorad. Pixel signal intensities were quantified using ImageQuant TL array analysis software (GE Healthcare).

### Supplementary Information

**Supplementary Material 1.**

**Supplementary Material 2.**

## Data Availability

All the data and material presented in the article are available from the corresponding author upon reasonable request.

## Abbreviations

CP: Coat Protein
CPMV: *Cowpea mosaic virus*
CterPSA: Carboxy terminal part of the PSA
CterPSA_spe_: CterPSA specific
dpi: days post infiltration
GAPDH: Glyceraldehyde-3-phosphate-deshydrogenase
HC: Heavy Chain
LC: Light Chain
mAbs: monoclonal antibodies
*Nb*EIF1-α: *Nicotiana benthamiana* Eukaryotic Translation Initiation Factor 1-α
ORF: Open Reading Frame
polyA: polyadenylated
PSA: Promastigote Surface Antigen
PVX: *Potato virus X*
q-RT-PCR: quantitative Reverse-Transcription PCR
RdRP: RNA-dependent RNA polymerase
RT-PCR: Reverse-Transcription PCR
RYMV: *Rice yellow mottle virus*
TMV: *Tobacco mosaic virus*
