# Supplementary material for: Rice yellow mottle virus is a suitable amplicon vector for an efficient production of an anti-leishmianiasis vaccine in Nicotiana benthamiana leaves
Source: BMC Biotechnol. 2024 Apr 24;24:21. doi: 10.1186/s12896-024-00851-8 (PMC11044499; doi:10.1186/s12896-024-00851-8)
Supplement: Supplementary file 1 — Supplementary Material 1. [file 12896_2024_851_MOESM1_ESM.docx]

**Additional file 1**: Sequence of the designed RYMV_Mg1_ΔP1ΔCP/CterPSA construct.

*Kpn*I and *Pst*I are noted in italic. CterPSA sequence is noted in bold characters.

*GGTACC*GTACCCCTACTCCAAAAATGTCAAAGATACAGTCTCAGAAGACCAAAGGGCTATTGAGACTTTTCAACAAAGGGTAATTTCGGGAAACCTCCTCGGATTCCATTGCCCAGCTATCTGTCACTTCATCGAAAGGACAGTAGAAAAGGAAGGTGGCTCCTACAAATGCCATCATTGCGATAAAGGAAAGGCTATCATTCAAGATGCCTCTGCCGACAGTGGTCCCAAAGATGGACCCCCACCCACGAGGAGCATCGTGGAAAAAGAAGACGTTCCAACCACGTCTTCAAAGCAAGTGGATTGATGTGACATCTCCACTGACGTAAGGGATGACGCACAATCCCACTATCCTTCGCAAGACCCTTCCTCTATATAAGGAAGTTCATTTCATTTGGAGAGGACAATTGAAGCTAGGAAAGGAGCATATTGCGAAAGCGATCCCTCCTTCCGACGAACAATTGTATCCACGCTGCTACGTGTATGACACGGGCATGCTTTTGAGGGAACACTCTGCCGACATCCAAGGAGCGGTCGTGGTTCAGACTAACACGGGATGGGCTCTTCTGTTGTTGGTCGCTTTCTCACTCGCACCAAGGAAACGACGAGGGTGGACTCCAACCTCCTCATCGTCTTGGCTGGGTTGATAGCGGCCGCGATAGTCTCAGAGCGCCTGCCCGTGACGGCGTCATTGTGGGCAATCCCCTCAGCTATCATAGCAAACTGGATCGTGCTGTCAGCTCACGAGAGCTTCAGCCGGTTCGTTGAGGGGGTTGAGATTGAGCCTATGTCCACGCTTCGGTATGGGAAGGTTCAGTCTGCTCCCCGGTTTGATCCCTCCCGGGGTTATGTGGTCGATGTTTCGTACAACGGCCACGTGATTCCGGTGATATTGGACTTCACCACGACTACGGCCCTGTCGGTTCCCCAAAGAGTAAACCCTGGGGTCTCGATGGAGGCTAGTCGTGGGGGGCTCCCACCGACGTCCGTCAAACTCGAAGATGTGCCGCCGAGCGTTGTGGTTTTGTACCACGACTCTGTCAGGCTAGGCCTTGGAACTAGGGTGCGAACTCCCACGGGTCGTGATTTGCTCATGACCAATCACCATATCGCTGCTCTAGAGCCCAATGGCATCGCGTATAAGGGTCACCTTAAGAAAGTGACTCTAGACGCGCCAGTCATCGCATGTGACCATCCACACATAGACTGTGCGTTTTACGAGGTGCCTCCCAAGATCTGGTCTCTCTTGGGGGTCAAGTCTGCCAGTTTGAAACCTTTGGTTAAACAGACTGCGGTGTCACTCTTCGGAGGCTCGTCATCCACTGACTTCTCGAGTTGTGTTGGGATAGCCCAGATTGGGGATAATCCATTCCTGATAAGGCATCAAAGCACAACTTGCAGTGGCTGGTCGGGCTCCCCGCTCTACCACAAAGGTTGCGTGGTTGGCTTACATATAGGTGCTGCGGATGGTTATAATGTGGCATCTAATGTAGCCTGGTACTTCCATACTTTCAAAAGGGATGTTGTTGTTGAGTCTCCATTCGAGATTTACGGCAAATTCCGGGAAGCAAACTCTGAGGAGTATGACGAAAGTCTGCGCCATGGGGTGGAATATGCGGAGTACGATTTCTCTGGTGACACAATCCGGGCGTCTTCCAACACCTGGGTGCGTGAGAGAGAGAGATACCACGCTGAGGAACGTCGTAAGTCCGGCCAGCTTAGCTGGGCAGATCGTTTTGGTGACGACAGTGGCGAGGATGTTGATATCGAGACATCGCATCCCGTAGCACCGTCAATATCTAAAACGCGGCAGAAACGGTCAAAGCGCGTTGAGCAGTTCGTCGACGCGGCTTCTGAGTGCTCCTTCTCGTTCGAGTCAGCTCACGAGGGGATTGTGCCAGAGACCTCAGCTTATGACCACGTTCCTTTAAACTGCCAGGGGGCGGGCTCGAGCCTTCGGGCGAGTCCGCCCTTGGACGGCTTATCCAACTCGGAGAATACCGCTGGGACTCCCTCGGTGACCCCCTCCCTTCCGACGGAATGCCCTTCAGCTACGTTGGAAAATCGGGTGTCATCTTTGGAGAACATGCTGGGAAAAGTGTCTGCGCAGCTGTCAAAGACGCAATCTCAGTATTCCCAGATCTTGAAGGATTTGGCTGGCCTGAGAGGGGAAGTAAAGCAGAGCTTGACTCCCTCATCCTCCAAGCCGGCCGGTTCAACAGAACGGTATGTCCCCCCGGGCTCGCGCAAGCAGTCCAATCACTCCAAGAAAAGTACCCAAAAGTCCCCCCCAGGCGATGCCTCAGGGACGAGTGGAGGTTCGACGACATCTTCGACGAAGTCGAAAGAATCCTCTGCGAAACCGGCGAAGTGAACTCCGCTTCCTCGCCGGGGGTGCCGCTGACGGGCCTTGCCAATTCCAATGGTGAGGTACGGAGGCTCGCGAGAGATTTGGTATGTTTAGCTGTGGTGGAGCGATTAAATGCCTTAGCCTCGGTTGACCCCCGCCAGCATAACTGGACACCCAGGGAGCTGGTAGAGAAAGGTCTTTGTGACCCTGTGCGTTTGTTTGTCAAGAACGAGCCGCACCCACAGAAGAAATTGCGTGAACGCAGATTCAGGCTGATTTCTTCTGTTTCTCTAGTTGACCAGCTGGTGGAGCGGATGCTTTTCGGGCCGCAGAACAACACTGAGATTTCAACTTGGTGGCAGTGGCCTTCGAAGCCGGGCATGGGTCTGTTGACCCCTGAACAAATCCGTTTGGTGTGGGACGACGTGTTCCAGAAGCACCAAGCCCACCCTGCGGCTGAGGCAGACATATCGGGGTTTGATTGGTCCGTTCAGGACTGGGAGTTGTGGTCAGACCTCGCGATCCGTATAAACAGAGGGAATTTTCAGGGAAACCTCAGGAAAGCGGCTATCAGCCGTTATTACTGTTTTATGAACTCTGTTTTCCAGCTCTCAGATGGAACTCTCATCCAGCAGGAGTTACCCGGGCTTATGAAGTCCGGCTCTTACTGCACCTCATCCACCAATTCACGCATACGCTGCCTAATGGCTGAGCTAATCGGCTCGCCCTGGTGCATAGCTATGGGTGATGATTCGGTGGAAGGATGGATTGAGGGTGCCCAGTCCAAGTACGCCGCGTTGGGTCACACCTGCAAGGAGTACTACCCATGCAAGACCAAGGGGCGTGAGCTCCTGGAATTCAACTTCTGTTCACACTTGATCAGGAGGGGTCATGCTGAGCTCACTTCATGGCCGAAGGCGCTTTTCCGCTTTCTGTCGAGCAAGCATGAGGACTTTGAAGATCTGTGGGTTGAGCTTCACACGTGCGGGGTGTGGAGCAGGATCGAACGATATCTGCGTGGGATAGGGCGAGTCTCCCACAAAGATGGCCAGGAAGGGCAAGAAAATCAACTCCAACCAAAGCCAGCAAGGCAAGAAGAAGAGCCGACGTCCTCGTGGGCGTTCGGCGGAGCCCCAGCTTCAACGGGCTCCAGTGGCTCAGGCGTCCCGGATATCTGGGACGGTTCCTGGACCACTATCTTCTAATACCTGGCCGGTCCACTCCGTGGAATTACTATACGTACTAGTGAAG**GCGCTGTGCGTGCGTCGGCTGGTGCTGGCGGCGACCCTCGCCGCTGTGGTGGCGCTGCTGCTGTGCACGAGCAGTGCGCCGGTGGCGCGTGCTAAGGCCGGTCTCGTTGTGGAAATCGAGGATAAGCACACGGGCAACAGCTGCATTGCTGGTGCGGACTGCGCAACGACGACCACGACCACCACTGAACCCACGTCCACTGCGAGCCCAACAGCCACGCCTACCTCTGCCCCCGAGACGGAGTGCGAGGTGGATGGGTGTGAGGTGTGCGATGGGGACTCCGCGGCGAGGTGCGCCAGGTGCCGTGAGGGCTACTTCCTGACGGACGAGAGGACGTGCCTGGTGTACCGCGATCATCATCATCATCATCATCATCATCATCACTGA**ACTAGTCCGGATTCAATGGAGATAGGTATTGGCACCAGTGATGATCCACGGATTAGCTCAGAGATCGACTACCCTGAAAAGGATGTTGAGCGTTTGCGAACGCGGGCTGAGTCCTACGAGATGATACTGGCGCCTATCCCATGTGGGGAATCGCACCCACGTTTCAACCCAAAGCGATCCATACGCTGGATAGCGTTTACCTGGTTGGTTTCGGAGCTGCCAGGAGGACCTCTTCACACTCCGGATTCCACATTCTTTCCGAGAGAATTCTCGGGATGGGTGGGGGAGAAGCTTCGACGGATCCCGGTACGCTGAAATCACCAGTCTCTCTCTACAAATCTATCTCTCTCTATTTTCTCCATAAATAATGTGTGAGTAGTTTCCCGATAAGGGAAATTAGGGTTCTTATAGGGTTTCGCTCATGTGTTGAGCATATAAGAAACCCTTAGTATGTATTTGTATTTGTAAAATACTTCTATCAATAAAATTTCTAATTCCTAAAACCAAAATCCAGTACTAAAATCCAGATC*CTGCAG*
