# Supplementary material for: Rice yellow mottle virus is a suitable amplicon vector for an efficient production of an anti-leishmianiasis vaccine in Nicotiana benthamiana leaves
Source: BMC Biotechnol. 2024 Apr 24;24:21. doi: 10.1186/s12896-024-00851-8 (PMC11044499; doi:10.1186/s12896-024-00851-8)
Supplement: Supplementary file 2 — Supplementary Material 2. [file 12896_2024_851_MOESM2_ESM.pptx]

## Slide 1
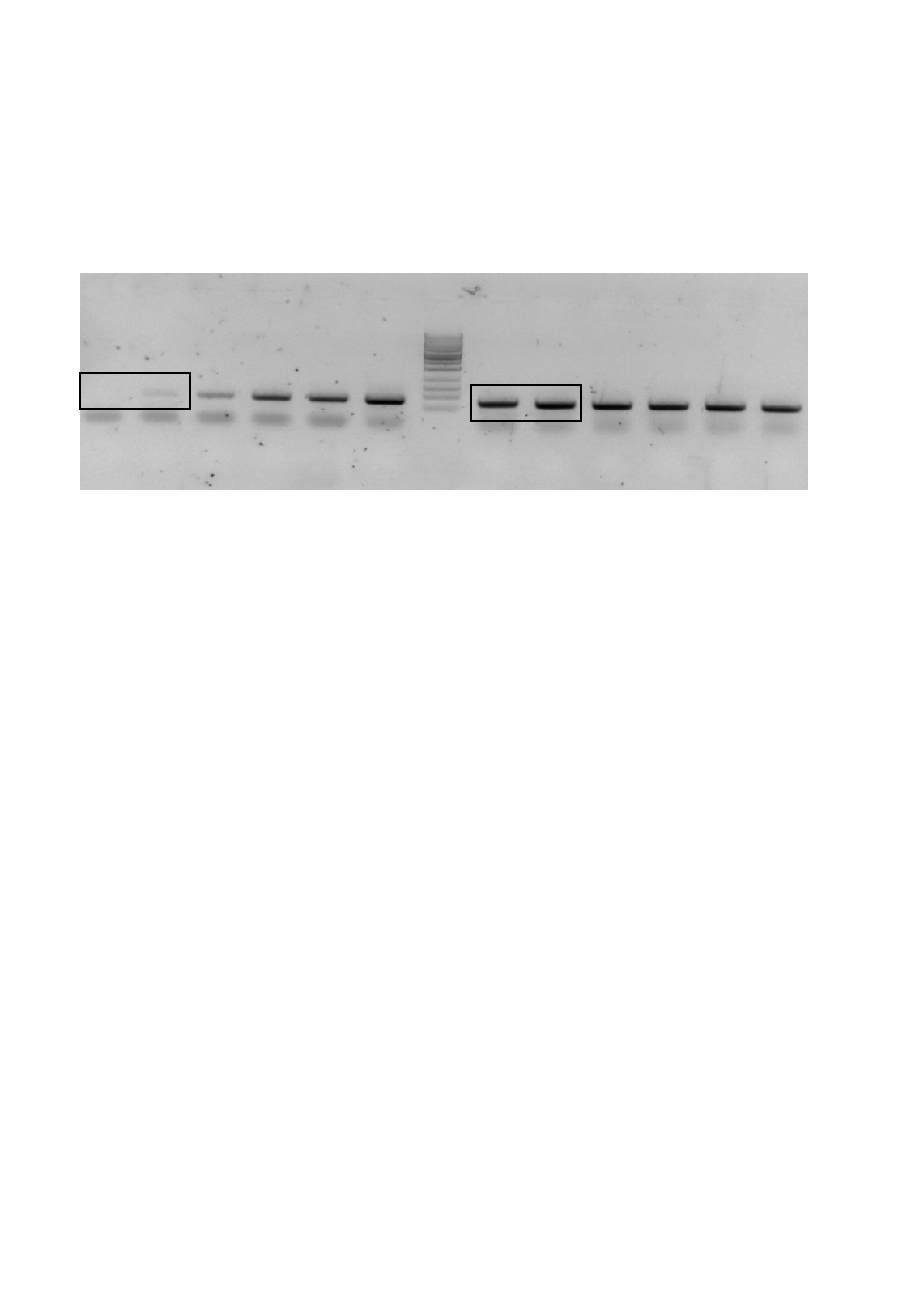

## Slide 2
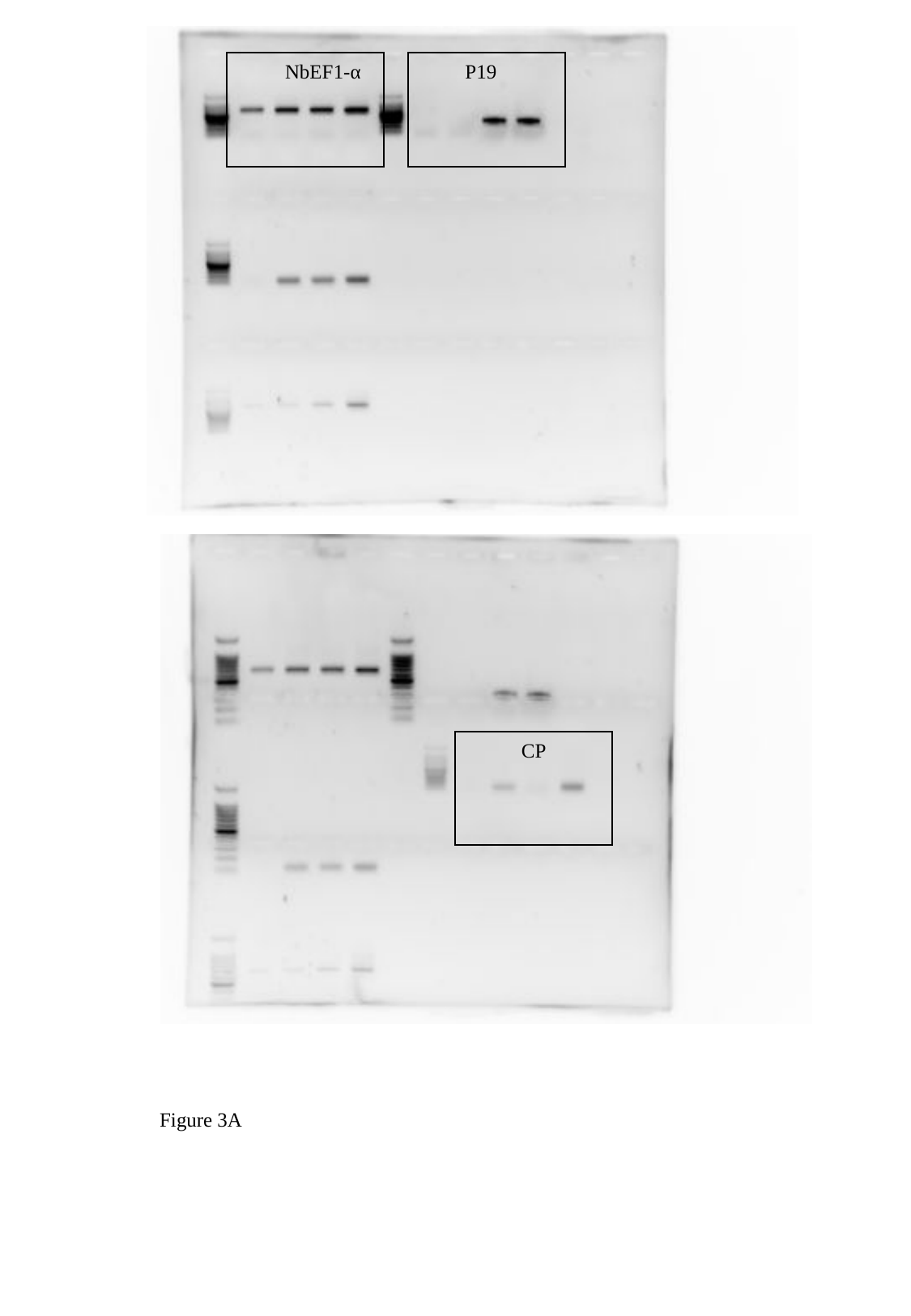

NbEF1-α
P19
CP
Figure 3A

## Slide 3
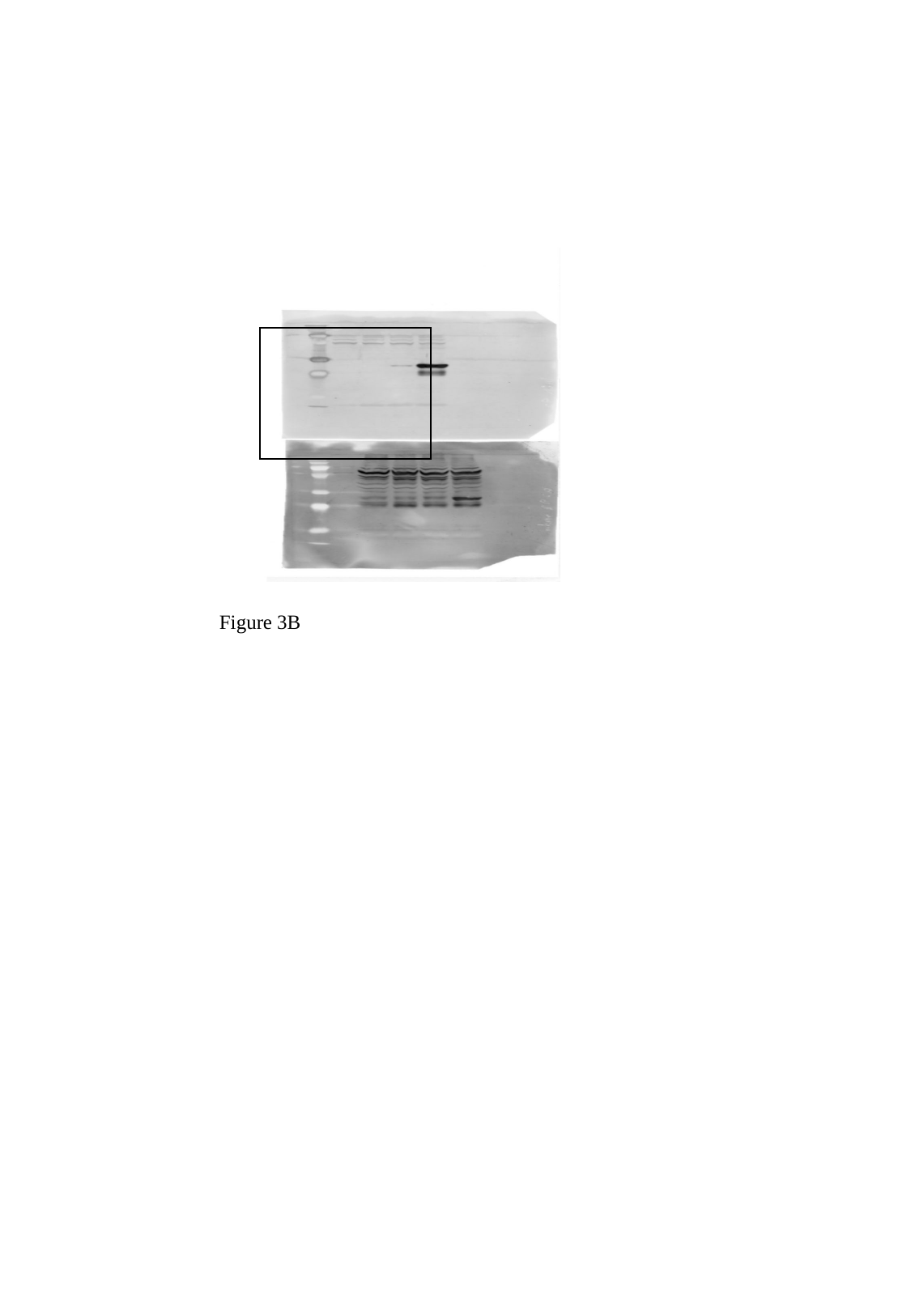

Figure 3B

## Slide 4
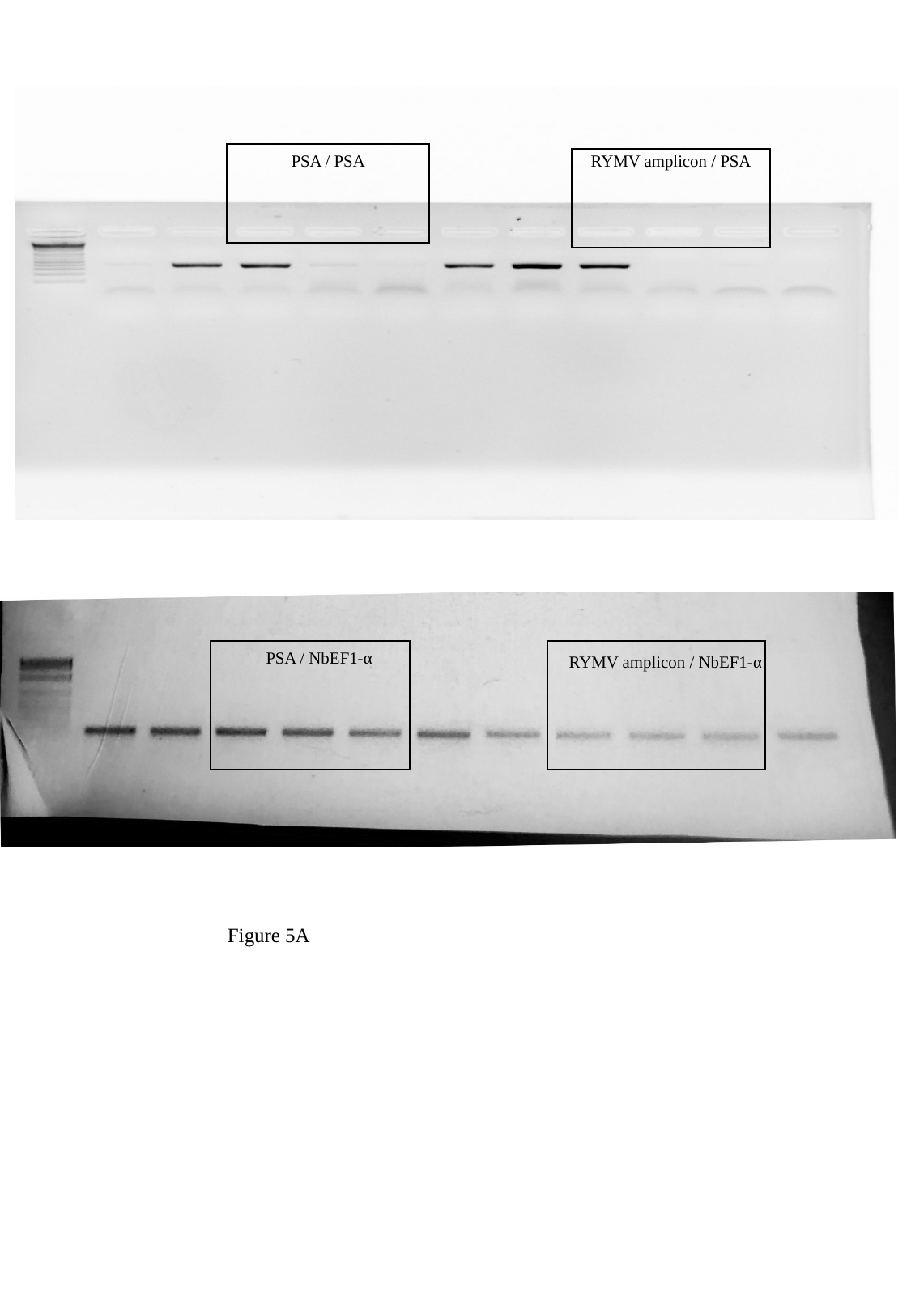

PSA / PSA
RYMV amplicon / PSA
PSA / NbEF1-α
RYMV amplicon / NbEF1-α
Figure 5A

## Slide 5
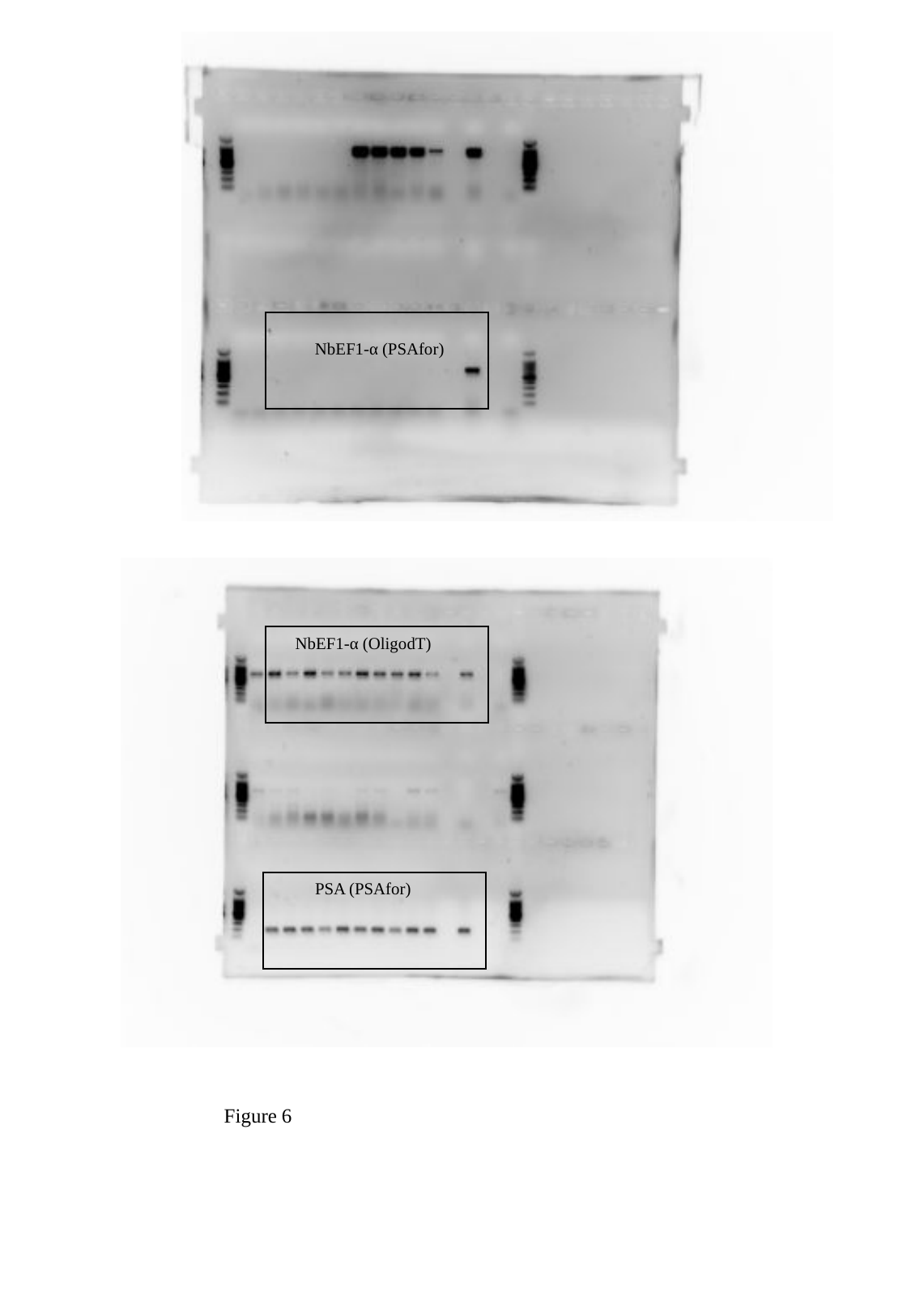

NbEF1-α (PSAfor)
NbEF1-α (OligodT)
PSA (PSAfor)
Figure 6

## Slide 6
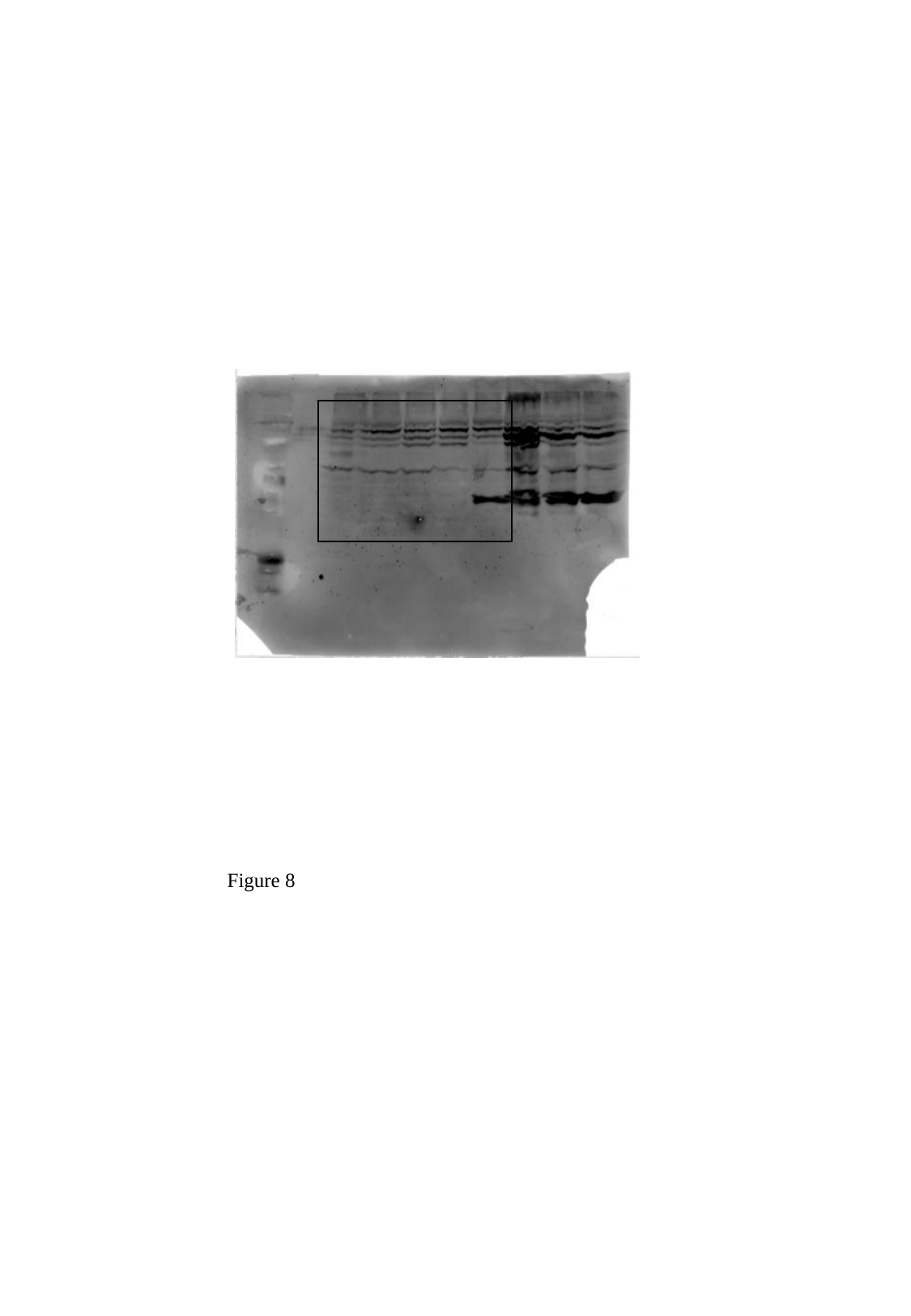

Figure 8
